# Advancing Colorectal Cancer Diagnosis with AI-Powered Breathomics: Navigating Challenges and Future Directions

**DOI:** 10.3390/diagnostics13243673

**Published:** 2023-12-15

**Authors:** Ioannis K. Gallos, Dimitrios Tryfonopoulos, Gidi Shani, Angelos Amditis, Hossam Haick, Dimitra D. Dionysiou

**Affiliations:** 1 Institute of Communication and Computer Systems, National Technical University of Athens, Zografos Campus, 15780 Athens, Greece; d.tryfonopoulos@iccs.gr (D.T.); a.amditis@iccs.gr (A.A.); 2 Laboratory for Nanomaterial-Based Devices, Technion—Israel Institute of Technology, Haifa 3200003, Israel; gidishani@gmail.com (G.S.); hhossam@technion.ac.il (H.H.)

**Keywords:** breathomics, colorectal cancer, volatile organic compounds, machine learning, artificial intelligence, automated diagnosis, validation, manifold learning, ONCOSCREEN

## Abstract

Early detection of colorectal cancer is crucial for improving outcomes and reducing mortality. While there is strong evidence of effectiveness, currently adopted screening methods present several shortcomings which negatively impact the detection of early stage carcinogenesis, including low uptake due to patient discomfort. As a result, developing novel, non-invasive alternatives is an important research priority. Recent advancements in the field of breathomics, the study of breath composition and analysis, have paved the way for new avenues for non-invasive cancer detection and effective monitoring. Harnessing the utility of Volatile Organic Compounds in exhaled breath, breathomics has the potential to disrupt colorectal cancer screening practices. Our goal is to outline key research efforts in this area focusing on machine learning methods used for the analysis of breathomics data, highlight challenges involved in artificial intelligence application in this context, and suggest possible future directions which are currently considered within the framework of the European project ONCOSCREEN.

## 1. Introduction

Cancer is a group of complex diseases linked to abnormal cell growth with devastating consequences for the patient. It ranks as a leading cause of death and a profound barrier to increasing life expectancy worldwide [1]. Detection of cancer in early stages along with timely and appropriate treatment is a critical component of reducing cancer-related mortality and morbidity [2]. Currently, there is a lack of reliable screening modalities for highly fatal cancers like pancreatic and gastric cancer [3]. Similarly, for highly prevalent malignancies such as breast and colorectal cancer (CRC), there is plenty of room for enhancing the existing screening practices. In particular, colonoscopy and fecal immunochemical test (FIT) are widely accepted as the cornerstones for the early detection of CRC [4]. During a colonoscopy procedure, early precancerous lesions can be detected and removed by a clinical expert. Nevertheless, colonoscopy is an invasive and costly procedure with low rates of compliance [5]. FIT serves as a complementary or alternative screening modality to colonoscopy for patients that decline the latter [4]; it is a non-invasive and low-cost test that serves as a widely adopted screening procedure for the large part of the average-risk population. Nevertheless, FIT shows modest accuracy in detecting CRC and advanced adenoma (AA), with sensitivity remaining under 70% and 50%, respectively [6]. Hence, CRC screening suffers from both low rates of adherence to the test (e.g., colonoscopy) as well as low detection rates (e.g., FIT).

In an effort to address the aforementioned problems, recent technological advances have brought up new novel non- or minimally invasive approaches such as breath, blood, and imaging-based tests [7,8]. In recent years, metabolomics has steadily gained momentum in various frontiers including disease detection and personalized medicine [9,10]. Breath volatolomics, also known as breathomics, can be seen as a branch of metabolomics, focusing on human breath. Breathomics studies volatile organic compounds (VOCs) and their metabolites that come from our respiratory system and internal organs. By simply exhaling air through a breathing device, it becomes possible to capture and analyze the profile of VOCs that are exhaled and present in the sample. Typically, a “breath biopsy” can be acquired in a non-invasive manner through the use of analytical methods like gas chromatography–mass spectrometry (GC-MS) or by utilizing sensors of various electronic nose devices [11]. Over two thousand VOCs have been reported to emanate from the human body [12], forming an inexhaustible treasure trove of biomarkers, which in turn have been linked to various diseases, including cancer [13,14]. Cancer cells undergo metabolic alterations which can result in the release of specific VOCs. For example, it has been shown that these cells tend to metabolize glucose via aerobic glycolysis rather than oxidative phosphorylation, an effect known as the Warburg effect [15]. Researchers posit the hypothesis that these VOCs are released into the bloodstream and eventually expelled through exhalation, passing through the endobronchial cavity [16]. A free web-based database, also known as the Cancer Odor Database (COD), contains comprehensive information about cancer-related VOCs, with its data being extracted directly from the scientific literature [17]. Another more general and recent database, the Human Breathomics Database (HBD) [18], contains comprehensive information about VOCs reported in 2766 publications. It provides biomedical information, underlying biochemical pathways and current scientific evidence regarding the association of each VOC with various diseases. In particular, research efforts on the determination of cancer-related VOCs have shown that some may contribute to more than five different cancer types [14,19]. For example, Nakhleh et al. utilized 13 VOCs for the detection and discrimination between 17 different disease conditions from 813 patients [14]. Despite the fact that breath analysis is still in early stages of development, analyzing breath composition holds significant potential for contributing to several subfields of cancer research such as detection [11,13], screening/monitoring [20], prognosis [21], and treatment response [11,22]. This review will focus on the relevance of volatolomics to CRC and recent theoretical and technological advancements derived from the field of breathomics in this regard.

GC-MS is undeniably the gold standard in breath analysis in terms of precision, as it enables separation, identification, and quantification of the different VOCs in the exhaled breath gas. Alternative ways such as sensor-based techniques have also been introduced with increasing interest [23]. Since GC-MS is resource-intensive, time-consuming, and requires special expertise, sensor arrays in the form of breathing electronic devices/noses (e-noses) constitute mobile, cost-effective, and user-friendly diagnostic alternatives that are capable of providing quick results. Studies employing e-noses and breath-based VOCs towards detection of CRC and AA are emerging at an increasing rate [24,25,26,27,28]. As a trade-off for their virtues, the latter detect mixtures of VOCs instead of identifying the actual mass of specific compounds. In other words, sensor arrays or e-noses are designed to imitate the human olfactory system with the use of chemical sensors [29]. Applications of e-nose devices in terms of odor perception are most often treated as black box models, focusing more on the accuracy of the task to be performed (e.g., diagnosis/monitoring) and less on understanding of how and why the subsequent results are derived [29].

While the use of chemical sensors holds the potential to revolutionize today’s medical diagnostics on CRC breath, it also faces significant challenges and limitations [30]. First and foremost, confounding factors such as age, diet, genetics, and smoking habits can introduce variability in breath composition, threatening with inaccurate results [14]. Second, timing and method of breath sample collection are critical considerations also, as exhaled breath profiles can change rapidly with fluctuations in blood chemistry [31]. Third, standardized protocols for uniform and repeatable breath sampling are imperative. Technical sensitivity, particularly regarding sensor responses to temperature and humidity, presents obstacles that necessitate controlled and sterile environments for analysis [32]. Fourth, data analysis also poses difficulties, with the choice of statistical methods, validation, and complex modeling needing careful consideration [33]. For example, the breath signature of CRC derives from statistical procedure; one has to seek differences between hundreds of VOCs that might be present. Searching for statistically significant differences between breath profiles of CRC patients and healthy controls, one needs to take into account the multiple comparison problem to ensure no false discoveries [33]. Today, breath-based VOCs reported as biomarkers for CRC detection exhibit a substantial amount of variation in the scientific literature [8,34,35,36,37,38,39,40,41,42,43,44]. Fifth, achieving strong predictive values for disease diagnosis and monitoring large-scale, multicenter clinical trials with blind validation are required [30]. Sixth, special emphasis on reproducibility and adaptation to real-world clinical conditions have to be given so as to formulate widely accepted technical and clinical standards in order to accelerate research and finally integrate breath analysis into routine medical testing. A study discussing technical standards and recommendations for sample collection and analytic approaches for lung disease can be found in [45]. Addressing these challenges is crucial to establishing breath-based diagnosis in the clinical practice.

In this article we particularly focus on how advancements from the field of machine learning (ML) and artificial intelligence (AI) can be used towards useful, reliable, accurate, and reproducible research towards breath-based diagnosis. It is the first review article that exclusively focuses on AI applications for CRC detection using breathomics; it aims to present the latest findings reported on the use of AI techniques and methods targeting breath-based VOCs for CRC diagnosis and has been performed in the context of the Horizon Europe project ONCOSCREEN (https://oncoscreen.health/, accessed on 10 December 2023). The project seeks to develop novel methodologies for cancer screening and early detection, ultimately aiming to enhance citizen awareness, participation, and adherence to relevant protocols. Among the various solutions proposed by the project, CRC diagnosis using breath-based VOCs is planned to be pursued utilizing both analytical and sensor-based methodologies. As a first step, a GC-MS instrument will be used for collecting prospective breath samples from healthy controls as well as CRC-diagnosed patients, thereby establishing a VOC signature database. Subsequently, a previously developed sensor array-based breath analyzer prototype [46], originally used for the detection of gastric cancer VOCs, will be modified based on the gas biomarkers defined in the GC-MS analysis phase. The basic analytical principle of the breath analyzer is based on the activity of a gold nanoparticle sensor array. This array was made of 8 different chemistries detecting the transient effect of the breath sample on the resistance of each of these sensors for 60 s. This led to the generation of 8 × 60 observations for each breath sample. The recent model has been upgraded to harbor 48 sensors resulting in 48 × 60 time points for each breath sample to enable a richer picture best analyzed by different AI frameworks. In our opinion, this mode of VOC mixture detection and labeling best resembles the principle of smell distinction existing in the mammalian olfactory bulb. Finally, an AI module of the device will be trained on electrochemical signal responses between healthy and CRC samples. The resulting new prototype will be tested prospectively for its ability to offer a portable and quick way of early CRC diagnosis. The project foresees a comprehensive testing and validation process, including a clinical validation study involving the enrollment of 4100 patients/citizens.

**Figure 1:**
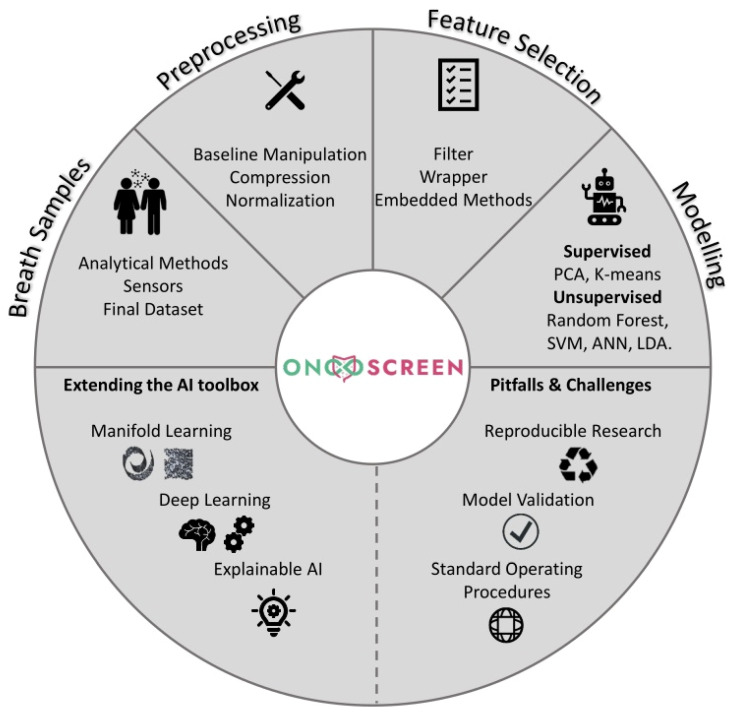
Schematic representation of the topics discussed within the scope of this study.

The rest of the article is structured as follows: we start with a brief introduction around CRC and then we explore its breath blueprint as it is derived from past studies. We then report the capabilities of contemporary diagnosis models for CRC and, subsequently, we dive into each step of a typical AI pipeline towards the diagnosis of CRC. We place special emphasis on identifying future challenges and considerations for the extension of the existing breathomics AI toolbox against CRC. We hope that this overview forms a basis upon which the community can further elaborate towards new advances taking into consideration the current challenges. A schematic representation of the topics discussed in this work can be seen in Figure 1.

## 2. Colorectal Cancer

Colorectal cancer can be categorized as colon or rectal cancer depending on where it initially develops. Despite their differences, these cancers are often grouped together [47]. The majority of colorectal tumors emerge from small clusters called polyps on the inner lining of the colon or rectum. The probability of a polyp transforming into cancer varies greatly depending on its type, with some of them never turning into cancer [48]. Polyps can be widely characterized as adenomatous, hyperplastic, inflammatory, or sessile serrated [49]. Hyperplastic and inflammatory polyps are frequently detected, with the adenomatous polyps being considered precancerous [50]. Due to a higher risk of developing into CRC, polyps characterized as sessile serrated are also regarded as adenomas [51]. Certain characteristics of polyps such as the size being larger than 1 cm, the segregation of more than three of them, or the detection of dysplasia after removal are linked with elevated risk of cancer development [52,53]. Over time, some precancerous polyps progress into cancerous growths within the walls of the colon or rectum [54]. The majority of CRC cases are adenocarcinomas originating from mucus-producing cells in the inner layer that play the role of lubrication and protection for the colon and rectum [55]. Of the adenocarcinomas, signet ring cell and mucinous cancers may have a less favorable prognosis [56,57]. Finally, less common types of CRC include carcinoid and gastrointestinal tumors, lymphomas, and sarcomas [58,59]. Typically, the progression of a polyp to a malignant state takes several years [54]. In many cases, patients do not experience symptoms until the cancer has already progressed to either an early or advanced stage of development [60,61]. Therefore, early detection, diagnosis, and staging by making use of diverse biomarkers are essential for effective cancer treatment [62].

## 3. Breath Blueprint as Biomarker for Early Detection and Monitoring of CRC

Scientific efforts towards the discovery of a CRC blueprint in breath began in the early 2000s [44,63,64]. Overall, CRC has been consistently linked with classes of VOCs such as alcohols, alkanes, aldehydes, and ketones [65,66], with the last two commonly found in cancer metabolism [67]. Multiple studies have tried to come up with a CRC breath profile [8,14,38,42,43,44,64]. Typically, these studies conduct VOC analysis in breath samples using GC-MS analysis and later check for differences between groups of CRC patients and healthy controls. If statistically significant differences are found in the levels of VOC prevalence between groups, then these VOCs are suggested as potential biomarkers for the disease. However, due to several differences in methodologies, technical equipment, pre-processing routine, sample sizes, or even different cancer stages (known to affect the VOC profile), a reliable and reproducible pattern of VOCs as biomarkers for general clinical practice is yet to become available. The list of CRC breath-based VOC biomarkers that have been reported in the scientific literature can be inspected in Table 1. Their underlying biochemical pathways and functionalities are further explored in [38,65,68].

Meta-analyses on the diagnosis of cancer focusing exclusively on breath-based VOCs have consistently shown optimistic results [68,69]. Specifically, on a systematic review and meta-analysis on different cancer types, Hanna et al. reported that despite a substantial variability among 63 studies, the pooled sensitivity reached 79% along with a pooled specificity of 89%. Xiang et al. further ratified previous results by focusing exclusively on gastrointestinal cancer and CRC, thus reporting pooled sensitivity of 85% and pooled specificity of 89%. They both concluded that while breath-based VOCs have the potential for clinical screening, standardized tools and protocols have to be introduced in an effort to mitigate the heterogeneity in the discriminatory VOCs reported and the subsequent diagnostic metrics. Finally, a more recent meta-analysis [23] considering 52 studies and 3677 patients with cancer, including CRC, reported 90% sensitivity and 87% specificity. The study exclusively focused on the diagnostic power of e-noses and though the authors reported optimistic results, they stated that most of the studies considered involved a small sample size and poor standardization.

Apart from meta-analyses relying only on breath-based VOCs, there have been some studies considering VOCs coming from different sources, such as fecal and urine VOCs. That being said, according to a meta-analysis on CRC screening using VOCs from different sources [70], the authors considered 10 studies spanning from 2012 to the end of 2019. The reported pooled sensitivity and specificity were found to be 82% and 79%, respectively. The results suggested that VOCs can be considered a stable and robust tool for CRC screening but not as a single and exclusive diagnostic test. Interestingly, VOCs associated with breath exhibited higher sensitivity and specificity than their counterparts (e.g., VOCs from feces and urine). Another meta-analysis from Wang. et al. [71] focused on diagnosis of neoplasms of the digestive system including CRC, using VOCs from different sources. Specifically for CRC, the authors included 16 studies (out of a total of 36 with over 3000 participants) and reported 84% pooled sensitivity and 82% specificity. Remarkably, the authors reported that breath-based VOCs behaved better (i.e., in terms of diagnostic metrics) than VOCs from other sources. Specifically, by considering only breath-based studies, the reported pooled sensitivity and specificity were both 87%. In Table 2 we present sensitivity and specificity rates reported in studies using breath-based VOCs as biomarkers for diagnosing CRC.

## 4. Applications of Machine Learning in Exhaled Breath Analysis: The Case of CRC

Machine learning (ML) can be seen as a part of AI that revolves around the development of methods aiming to enable machines to learn from data. Applications of ML span various domains, including medicine and disease diagnosis, particularly in situations where traditional algorithms are impractical and/or insufficient. Roughly speaking, ML approaches can be divided into three broad categories of learning, namely supervised, unsupervised, and reinforcement learning. The main difference between the supervised and the unsupervised learning paradigm lies in the fact that in the first case the computer is learning from labeled examples with known inputs and desired outputs. On the other hand, unsupervised learning tries to discover inherent structures from unlabeled data without explicit guidance, typically with consideration of some measure of similarity between the data entities. Finally, reinforcement learning typically involves an agent that is learning to make decisions through trial and error, receiving feedback in the form of rewards/penalties. Through an iterative process, long-term cumulative rewards are maximized based on the observed outcomes. For the rest of this review, we will focus on the supervised and the unsupervised learning paradigms.

In the following, we sketch the main pillars of the standard ML methodology in VOC analysis. Typically, the analysis pipeline starts with data acquisition, either with an analytical or a sensor-based method (for their relative advantages and disadvantages see Section 1). It continues with the pre-processing and feature extraction steps. Preprocessing is a stage where we transform the raw data into a comprehensible format to augment the downstream analysis. Feature extraction is the process of generating new values (features) from the initial measurements that are informative, non-redundant, and aid in the subsequent learning, ultimately leading to useful and informed models. The next step usually includes feature selection, a level of analysis where we choose the most discriminatory features/biomarkers present in our dataset, towards parsimonious modeling to enhance predictive and generalization capabilities. Then, we proceed to the actual modeling for discrimination between patients and healthy controls in a supervised/unsupervised manner. Finally, model validation takes place to assess the model’s performance and confirm the usefulness of the model in real world applications.

In Table 3 we present the key characteristics of the pipeline used in studies considering breath-based VOCs towards the diagnosis of CRC.

### 4.1. Pre-Processing and Feature Extraction

Pre-processing is the initial stage of processing raw data. Roughly speaking, pre-processing involves transforming raw data into a format that is comprehensible and augments the performance of the following steps. The most commonly applied strategies can be broadly categorized as baseline manipulation, compression, and normalization transforms [74]. The baseline manipulation refers to the transformations that attempt to correct for the baseline of the signal with the aim of suppressing the effect of sensor drifts (e.g., signal slowly deviates independently of the measured property due to changes in temperature, electronic aging of components, etc.). Compression transformations address the problem of dimensionality, effectively reducing the number of measurements trying to optimize the trade-off between an accurate representation and a reasonable size of the final dataset. Normalization transformations are commonly applied to smooth variations between sensors, such as for example an inherently higher signal magnitude of some sensor over the others. Other forms of preprocessing align more to the quality assurance of the data. Such procedures include the removal of artefacts, suppression of noise, and handling the missing values via imputation [34]. These types of transformations can also enhance the performance of ML algorithms in terms of faster convergence in the optimization process, robustness of results, and accuracy [75].

In the context of ML and pattern recognition, feature extraction plays a crucial role as it involves taking an initial set of measurements and generating new values (features) that are informative, non-redundant, and aid in the subsequent learning and generalization processes. Despite the fact that it is very difficult to categorize the different families of methodologies, feature extraction methods can be divided into three main groups, the piecemeal, the curve fitting/statistical measures, and the transformation-based techniques. Regarding the piecemeal features, these are the features that are directly computed on the sensor’s response, including first and second derivatives which can be translated as the reaction rate of the sensor and the acceleration, respectively. Other features in this category involve measures such as the computation of maximum value, the rising and the falling slopes during steady state, transient response, and others [76]. In the case of the curve fitting methods, we actually fit a model on the sensor’s response in order to measure specific model parameters [77]. Models that are commonly used for fitting purposes include polynomial function, exponential, and auto-regressive models. Here, we could also consider statistical measures that are computed directly on the distribution of the sensor-response such as mean, median, skewness, kurtosis, etc. Finally, there are transformation-based methods involving the conversion of our signal to the frequency domain such as the Discrete Fourier Transform (DFT) or the Discrete Wavelet Transform (DWT), which combine the virtues of DFT but also preserves temporal information. An example of successful application of DWT can be found in [78]. The authors pre-processed their data using DWT and later applied Principal Component Analysis (PCA) trying to discriminate between the different odors.

Since the preprocessing routine plays a significant role in the subsequent steps of a long pipeline, it is particularly important to be reported in detail, so as to have a common ground and the diverse results reported by different authors can be compared. In Table 3 the reader can inspect the different preprocessing pipelines used in studies considering breath-based VOCs towards CRC diagnosis. It is remarkable that for 5 out of 14 studies included in Table 3, we did not manage to find comprehensible information on the preprocessing pipeline applied by the authors.

### 4.2. Feature Selection

Distinct from feature extraction, feature selection focuses on choosing a subset of existing features rather than creating new ones. Overall, feature selection plays a crucial role in enhancing the efficiency and effectiveness of data analysis. It aims to simplify models, reduce computational times, increase accuracy, robustness of learning, and enable the interpretability of the final model (such as suggesting a few biomarkers that are probably connected to a disease) [79]. The rationale/hypothesis behind feature selection is that the data usually contain redundant and/or irrelevant features that can be eliminated without significant information loss. While the simplest approach involves testing each possible subset to minimize the error rate, this exhaustive search is computationally impractical for large feature sets. The feature selection algorithms are divided into three broad categories: filter, wrapper, and embedded methods [24].

Filter methods are computationally efficient and capture the usefulness of the features based on statistical measures such as correlation but are not tuned to a specific model. For example, there is no complex predictive model involved and, thus, no parameter selection is needed. Instead, these methods may measure the degree of association between the target and the independent variables (e.g., in our case, how the healthy population differs from the diseased; with respect to which features?). They tend to produce more general feature sets but they usually score lower in prediction performance than wrappers or embedded methods. Examples of such methods and their applications include the Analysis Of Variance (ANOVA) [36,40], Welch’s *t*-test [8], and the Mann–Whitney U test [41,43]. Wrapper methods use a predictive model to score feature subsets. Each subset is used to train and test a model with its error rate, producing a final score. While computationally intensive, wrapper methods are most likely to produce better results than filter methods. Examples and applications include stepwise selection [38,72], the recursive feature elimination process [21], and the evolutionary search [72]. Embedded methods incorporate feature selection as part of the model construction process. Examples of such methods and their applications include the Least Absolute Shrinkage and Selection Operator (LASSO), which penalizes regression coefficients using L1 norm regularization [37,80], ridge regression (L2 norm regularization), Elastic Net regularization [36] (which combines L1 and L2 norm regularization), and Random Forests which utilize the Gini impurity index or information gain/entropy for ranking features by relative importance [36,39]. These methods offer a balance between filters and wrappers in terms of computational complexity.

### 4.3. Modeling and Classification

The most relevant features considered in the previous steps constitute the final feature set that is naturally used for modeling and classification. This can be performed in either a supervised or unsupervised manner. The latter does not involve class labels and tries to blindly find statistical similarities between data points with the ultimate goal of finding associations or distinct clusters of similar data points in a sample. A popular algorithm for data-driven modeling in an unsupervised manner is the Principal Component Analysis (PCA) [73,81], which can be used in conjunction with the K-means clustering algorithm for classification purposes. K-means have been utilized in cancer research including CRC [82,83]. On the other hand, supervised learning uses class labels as ground truth to train a model performing over specific tasks. Applications of supervised learning include algorithms such as Random Forests [35,39], Support Vector Machines (SVM) [36], Logistic Regression [37,38,40], Artificial Neural Networks (ANN) [26,27,41,43,73], and Linear discriminant Analysis (LDA) [8]. Despite the fact that supervised learning is more extensively used in disease diagnosis, unsupervised learning is particularly helpful for visualizing the data through clustering and gaining insights into the nature of a particular phenomenon or disease.

It should be noted that every algorithm has its own strengths and weaknesses and no consensus on the general use of specific algorithms exists in the literature. The algorithms can be further divided into linear and non-linear. The non-linear algorithms assume a nonlinear relationship between the target variable and predictors used for classification. In the linear case, separation between groups can be achieved through a linear combination of the explanatory variables and, in simplifying terms, this can be thought of as a straight line on a 2D plane. The nonlinear case involves nonlinear relationships among predictors to achieve separation. Despite the conceptual superiority of non-linear algorithms and the often better predictive performance, they are more complex and therefore often hinder the ability to interpret the final model [84,85]. Explainability in the context of AI applications refers to our ability to explain why and under which circumstances a decision is made by a trained model. For example, in a clinical setting, medical experts are interested in the clinical inference, which in turn plays a crucial role in the diagnosis, staging, or following of a specific curative treatment [86]. Finally, other factors to consider when it comes to the selection of a specific algorithm are the computational complexity and the proneness to overfitting which must be assessed thoroughly through the process of model validation [87].

### 4.4. Model Training and Validation

Model training and validation can be seen as two distinct parts towards modelling. Training refers to the process of fitting the best combination of parameters to the model using a training set, while validation refers to the evaluation of performance using a validation set (e.g., to tune hyperparameters) and a test set. In practice, in order to develop an AI model, multiple models are fitted and we ultimately choose the best candidate (with specific parameters and hyperparameters) judging by its performance on the validation set. If we incorrectly assess a model’s performance, then we might choose a useless configuration. Naturally, the validation strategy affects both the internal parameters (such as weights and biases, which are parameters automatically derived during the training process) and hyperparameters (which are essential for optimizing the model and are externally set by the researcher) of a model. In cases where we consider a feature selection process during training (i.e., candidate models consider different feature sets), insufficient validation may affect the suggested biomarkers [88,89,90]. That said, model validation is involved whenever training occurs, either only to estimate (e.g., in an unbiased manner) prediction performance (e.g., accuracy, sensitivity, specificity, etc.) or to tune parameters with respect to them. At last, the final error estimate is obtained when the best candidate model is finally applied on unseen test samples. Of course, we expect that the model scores more or less the same as when applied to the validation data. In a case where the test error is much larger than the validation error, overfitting can occur. Hence, model validation gives us a hint of the expected test error while testing aims for an unbiased estimate of the model’s performance in a real clinical setting, battling the well-known phenomenon of overfitting [88,91]. The test dataset should be independent of the training and validation sets.

Commonly applied validation strategies for both tuning AI algorithms and estimation of performance are the Hold-out strategy (e.g., splitting into train, validation, and test set) and CV. The Hold-out strategy splits the data into training, validation, and test sets and follows the procedure described above. CV divides data into k subsets. The model is trained and evaluated repeatedly k times, each time using different subsets leaving one subset out for validation and the rest for training purposes. Next, performance metrics are obtained and averaged with the aim of providing estimates of the model’s prediction error. When k matches the number of samples in the data set, the method is called Leave One Out Cross Validation (LOOCV). The main difference between the two approaches is that cross-validation utilizes the entire dataset enabling all data to be incorporated in model training and validation. CV may help in reducing the variance in model performance estimates induced by a specific split into training and testing data [91]. The last step is again to test the best candidate model (e.g., based on CV score) on an unseen and independent testing dataset.

Finally, the validation strategy can be characterized as internal or external depending on the cohorts/datasets used for validation. Internal validation refers to the validity of the model inside a single cohort, while external validation refers to the validity of the model spanning external cohorts. External validation is much more powerful than internal in the sense that the model is capable of performing as intended even when there are substantial differences among data sources. For a diagnosis model on CRC, external validation would mean to test the ability of the model to diagnose the disease in cohorts of hospitals in different countries, for patient populations with different demographics, etc. Here, we have to note that none of the 14 studies included in Table 3 used an external dataset to validate findings. Moreover, most of the time the authors reported the validation error (e.g., mainly due to the fact of using limited samples) since the cross validation is applied on the entirety of the available datasets (Table 3).

## 5. Future Considerations: Extending the AI Toolbox towards Disease Diagnosis

This section delves into future considerations that extend the AI toolbox currently in use, regarding breath-based diagnosis of CRC. These forward-looking directions are framed within the context of the ONCOSCREEN project’s ongoing advancements and developments. We explore three key axes: manifold learning, deep learning, and explainable AI, each representing a critical dimension in the quest to enhance the understanding and performance of the contemporary models on diagnosis of CRC.

Over the past years, linear data-driven approaches such as PCA and LDA have become part of the conventional breath analysis research towards preprocessing, dimensionality reduction, visualization, and modelling in terms of a few “dominant”, discriminatory variables [8,73,81]. In the context of dimensionality reduction, nonlinear alternatives of the aforementioned methodologies have been introduced like the kernel Principal Component Analysis (kPCA), ISOmetric feature MAPping (ISOMAP), Locally linear Embedding (LLE), and Diffusion Maps [92,93,94,95]. Originating from the field of manifold learning, the fundamental assumption is based on the manifold hypothesis, suggesting that high-dimensional data often lie on or near a lower-dimensional manifold within the high-dimensional space. In simpler terms, it is assumed that the data can be effectively represented in a lower-dimensional space taking into account nonlinear (or locally linear) measures (such as the geodesic distance between data points [90]) of similarity between data points. By exploring and leveraging the intrinsic structure of high-dimensional data, one can enhance not only the diagnostic capabilities of a model, but also uncover subtle patterns and relationships within complex datasets. For example, given a number of features resembling either a sensor’s resistance or the levels (e.g., abundance) of VOCs in the breath samples, we can find eigenvectors that capture non-linear combinations of our initial feature set and tentatively follow the intrinsic geometry of the underlying manifold. This may allow for visualization and exploration of non-trivial and subtle properties. Such techniques have been successful in various fields where big, complex data and non-linear phenomena are involved. Applications include the diagnosis of schizophrenia with the use of functional magnetic resonance imaging data [90,96], classification of images of handwritten digits [92], forecasting of brain signals [97] and financial time series [98], bifurcation analysis from spatio-temporal data produced by lattice Boltzmann simulations [99], and others. Specifically, Gallos et al. [96] used a variety of manifold learning techniques to construct (embedded) brain connectivity networks (e.g., by mapping correlation matrices in the low dimensional space prior to network construction), utilizing graph theoretic measures towards diagnosis of schizophrenia. Diffusion Maps outperformed their linear counterparts in terms of diagnostic capability. In a follow up study [90], ISOMAP was also applied to demonstrate that learning and feature selection on the low dimensional space was again beneficial in simplifying and raising the predictive performance of the model, ultimately leading to the discovery of a few informative biomarkers for the disease.

Beyond the classic ML methodologies, a subset of ANN-based frameworks also known as deep learning (DL) led to breakthroughs in several fields such as medical image processing [100] and medical diagnosis [101]. DL’s ability to process large scale data enables the analysis of raw data even without pre-processing, frequently with high precision [24,102]. In particular, applications on breath-based VOCs towards cancer diagnosis have been introduced [103] and frameworks have been suggested. These include time series stemming from e-nose devices [104]. Specifically, for CRC, the efforts have targeted colonoscopic [105], endoscopic [106] and histopathological [107] images, mostly by using Convolutional Neural Networks (CNN), an architecture designed for analyzing visual data. These types of ANNs utilize convolutional layers to automatically extract meaningful features and have achieved remarkable success in computer vision tasks such as image recognition and object detection. A review can be found in [108]. Within the context of DL, different architectures exist. These include Autoencoders, which utilize a bottleneck layer to extract meaningful features or reduce the dimensionality [103]. An additional method is Recurrent Neural Networks (RNN), which consist of recurrent connections to preserve information across time steps, hence allowing for them to capture temporal dependencies. The latter are also suitable for time series analysis and have been applied towards the optimization of e-nose systems [109] and odor classification [110]. Despite their merits, DL frameworks have their own limitations and challenges. Typically, they need vast amounts of data in order to reach their full potential, they are computationally expensive, have elevated probabilities of over-fitting, and are accompanied by high complexity and low interpretability. A comprehensive discussion can be found in [111].

In an effort to address the interpretability of the AI models, remarkable advancements have been made towards the establishment of tools and frameworks that provide understandable explanations regarding outputs and decisions made by the AI models [112,113,114]. In the literature, the predominant terminology for this field is Explainable AI (XAI) [115]. A characteristic framework is the so-called Shapley additive explanations (SHAP), an approach originating from game theory that attempts to explain the output of AI models. The Shapley values can be used in a model agnostic way [116], thus serving as a useful tool that may accompany various machine learning algorithms. Conceptually, it can be regarded as an extension of the Local Interpretable Model-agnostic Explanations (LIME) approach [117]. In simple terms, the absolute Shapley value reflects how each feature contributes to the final outcome as it is derived from an AI model [113]. However, only a few studies have considered such frameworks on CRC [112]. For example [112], tried to classify CRC patients based on the gut microbiome, and managed to both find CRC-associated bacteria and explore subgroups of CRC patients based on PCA that was imposed on SHAP values. Other frameworks include Gradient-weighted Class activation mapping (Grad-Cams), and these can be applied to CNN-based models to provide transparency and visual explanations [118]. Applications on the diagnosis of CRC include the diagnostic evaluation from colonoscopy images [105] and, more recently, CRC diagnosis and grading utilizing histopathological images [107]. Since this methodology is compatible with CNN-based models, it is natural that it can also be used on time series data employing 1D CNN models [119,120] or even encoding (multivariate) time series data (e.g., one univariate time series per sensor of an e-nose) into two-dimensional images (e.g., a correlation matrix or a dissimilarity matrix in general) towards classification/diagnosis (e.g., CRC patients and healthy controls) via pattern recognition [121].

## 6. Challenges and Pitfalls in the Use of AI Modelling towards Diagnosis

While the use of AI and especially ML towards diagnosis has been predominant in the past years, one has to put a significant emphasis on model validation to ensure their model generalizes into new unseen data [91]. Estimation of the true capabilities of the contemporary AI models should not be taken lightly. First of all, in cases where the dataset is imbalanced, something very common in disease diagnosis, splits of the dataset (e.g., into training and test splits) should be performed in a stratified manner. This means to practically keep the same percentages of classes in each split. Second, in order to avoid data leakage, transformations such as standardization of data or PCA should consider only the training data. This applies because we do not want our model to be trained using information contained on the unseen test dataset, for this would strongly bias our estimates of performance and likely the true capabilities of the model. Third, it is of paramount importance that wherever a CV procedure is used (with the exception for LOOCV), the estimated performance of the model should be reported in terms of average and standard deviation, which reflect the variability of the model’s performance [89]. For even better/refined estimates of model performance or parameter tuning, repeats of CV can also be beneficial [122]. Fourth, even a loop of CV may not be enough to come up with an unbiased estimate of the model’s performance, especially when feature selection is taking place at the same time inside the same loop [90,123]. Fifth, feature selection should be used in conjunction with CV (especially when embedded methods are used), as the opposite has been shown to strongly overestimate model’s performance [122]. To put it simply, it is crucial that the feature selection process does not “see” all data and then use the optimal feature set to evaluate the performance of a model on the same set. Hence, it is strongly advised that the validation of AI models should take place in the form of a nested CV consisting of an inner loop for optimizing hyperparameters and an outer loop of CV for evaluating performance. Sixth, it is highly recommended to always test the final model on an unseen test dataset (i.e., that is not considered during the model construction phase) after estimating the predicted performance of using a CV scheme. Specifically, Varma et al. [123] reported that the difference between the single CV error estimate and the true error was in some cases greater than 20%, which can be dramatically significant, especially in cases where the classification rates are moderate. Finally, it should be made clear that a CV for assessing a model’s performance produces an estimation of prediction error and by no means can this be considered the true test error, which can only be inducted by using sufficiently large unseen test samples (i.e., external validation). This is a common misconception in the scientific community [122,124].

## 7. Conclusions

This article focused on AI/ML methods used for the analysis of breathomics data in the context of CRC. The needs for improved CRC screening and monitoring were highlighted in parallel with the reported shortcomings of the contemporary standard protocols. VOCs that have been identified as potential biomarkers in previous studies have been presented. Further, we presented the diagnostic performances of contemporary models along the AI pipelines. We explored the main steps of typical AI pipelines in breath analysis for both analytical and sensor-based techniques. The latter are promising methods holding several potential advances over analytical methods in terms of cost, time, portability, and ease of use. Next, we stated future considerations and challenges with a view on extending the AI toolbox that is currently used towards CRC diagnosis via breathomics. The review discusses new potentials in the use of AI, such as the applications of non-linear dimensionality reduction/manifold learning algorithms, DL frameworks, and XAI sets of tools. These tools can potentially enhance diagnostic performance, explore non-linear and complex relationships among features, and provide insights into a “finer” choice of biomarkers with contribution to diagnosis. Despite the optimistic results of breath-based diagnosis in terms of sensitivity and specificity, there is substantial variability among studies and a reliable device and/or pipeline is yet to be developed. In this direction, model training and validation procedures have to be strictly defined and the model’s capabilities need to be reported in terms of both internal and external validation. Finally, preprocessing pipelines should be reported transparently and in more detail towards reproducible research.

## Figures and Tables

**Table 1:** Biomarkers reported for CRC.

Technique	Sample Size	VOC	Reference
**GC-MS**	CRC (15); Controls (20)	Acetone; heptanoic acid; 2,6,10-trimethyldodecane;	Śmiełowska et al., 2023 [34]
**GC-MS**	CRC (30); Controls (84)	2-propenoic acid ethenyl ester; lactic acid; 2,4-dimethyl-pyrrole; p-menth-3-ene; 6-methyl heptane; 2,2,4,4-tetramethylpentane; 2-methylfuran; propyl pyruvate; and 2 unknown identified VOCs	Cheng et al., 2022 [35]
**GC-MS**	CRC (162); Controls (1270)	propyl propionate; dimethyl sulfide; 1-penten-3-ol; 3,4-dimethyl-1,5-cyclooctadiene; 2-propenyl ester of acetic acid; branched tetradecane; 2-methyl-2-propanol; 4-ethyl-1-octyn-3-ol;2,2,4-trimethyl-3-pentanol; cyclopropane; 2-ethoxypropane; 2-phenoxy-ethanol; heptane; branched tridecane;	Woodfield et al., 2022 [36]
**IMR-MS**	CRC (52); Controls (45)	Dinitrogen Oxide; Nitrous Acid; 1,3-Butadiene; Acetic Acid; Unknown identified VOCs (9)	Politi et al., 2021 [37]
**GC-MS**	CRC (83); Controls (90)	Tetradecane; ethylbenzene; 5,9-undecadien-2-one, 6,10-dimethyl (E); decane; benzoic acid; 1,3-bis(1-methylethenyl) benzene; decanal; unidentified compound; ethyl-1-hexanol; dodecane; ethanone; 1{[}4-(1-methylethenyl)phenyl{]}; acetic acid	Altomare et al., 2020 [66]
**SIFT-MS**	CRLM(51); Controls (54)	(E)-2-Nonene; acetaldehyde; triethyl amine	Miller-Atkins 2020 [39]
**SIFT-MS**	CRC (50); Controls (100) *	Propanal	Markar et al., 2019 [40]
**GC-MS**	CRC (71); Controls (89)	2-ethylhexanol; 3-methylhexane; 5-ethyl-3-methyloctane; acetone; ethanol; ethyl acetate; ethylbenzene; isononane; isoprene; nonanal; styrene; toluene; undecane	Nakhleh et al., 2017 [14]
**GC-MS**	CRC(65); Controls (122)	Acetone, 6 ethyl acate, ethanol, 4-methyl octane	Amal et al., 2016 [8]
**GC-MS**	CRC (48); Controls (32) **	1,2-pentadiene; beta-pinene; 2-methylbutane; 1-methyl-3- (1-methylethyl)benzene; 2-methylpentane; 1-(1-methylethenyl)-2-(1-methylethyl)benzene; 5-butylnonane; methylcyclopentane; undecane; cyclohexane; heptane; nonanal; methylcyclohexane; dodecane; 4-methyl-2-pentanone; decanal; 1-methylnaphthalene; 1-ethyl-1,2,4-trimethylbenzene; 1-octene 1-ethyl-2,4,5-trimethylbenzene; octane; 2,3-dihydro-1,6-dimethyl-1H-indene; 1,2,3-trimethylbenzene; 2,3-dihydro-4,7-dimethyl-1H- indene; 1,3-dimethylbenzene; 1,3-dimethyl-5-(1-methylethyl)benzene; 1,4-dimethylbenzene; 2-methylnaphthalene; propylbenzene;	Altomare et al., 2015 [41]
**GC-MS**	CRC (20); Controls (20)	Cyclohexanone, 2,2-dimethyldecane; dodecane; 4-ethyl-1-octyn3-ol; ethylailine; cydoctyimethanol; trans-2-dodecen-1-ol; 3- hydroxy-2,4,4-timethylpentyl2-methyipropanoate; 6-t-buty4- 2,29,9-tetramethyl-3,5-decadien-7-yne	Wang et al., 2014 [42]
**GC-MS**	CRC (37); Controls (41)	Nonanal; 4-methy1-2-pentanone; decanal; 2-methylbutane; 1.2-pentadiene, 2-metyipentane,3-methylpentane; methylcyclopentane; cyclohexane; methylcyclohexane; 1,3-dimethylbenzene; 4 methyloctane; 1,4-dimethylbenzene; a(4- methylundecane, rt = 11-3); b(timethyldecane, RT = 13-2)	Altomare et al., 2013 [43]
**GC-MS**	CRC (26); Controls (22)	1,10-(1-butenylidene)bisbenzene; 1,3-dmethy benzene; 1- iodononane; {[}(1,1-dimethyiethyl)thio{]}acetic acid; 4-(4-propylcyclohexyl)-40 cyano{[}1,10-biphenyl{]}-4-yl ester benzoic acid; 2-amino-5isopropyl-8-methyl-1-azulenecarbonitrile	Peng et al., 2010 [44]

Note: CRLM: colorectal cancer liver metastases; GC-MS: gas chromatography—mass spectrometry; SIFT-MS: selected ion flow tube mass spectrometry; IMR-MS: ion molecule reaction–mass spectrometry. * 50 of controls with normal LGI tract endoscopy and 50 found positive including inflammatory bowel diseases (IBD), diverticular disease, and polyps. ** Former patients found disease free after oncologic follow up.

**Table 2:** Reported sensitivity and specificity on diagnosis of CRC based on exhaled VOCs.

Technique	Sample Size	Sensitivity	Specificity	Reference
**Sensors**	CRC (105); Controls (186)	0.79	0.53	Poļaka et al., 2023 [72]
**GC-MS**	CRC (15); Controls (20)	0.94	1	Śmiełowska et al., 2023 [34]
**GC-MS**	CRC (30); Controls (84)	0.8	0.7	Cheng et al., 2022 [35]
**GC-MS**	CRC (162); Controls (1270)	0.79	0.86	Woodfield et al., 2022 [36]
**IMR-MS**	CRC (52); Controls (45)	0.96	0.73	Politi et al., 2021 [37]
**GC-MS + sensors**	CRC (82); Controls (87)	0.9	0.93	Altomare et al., 2020 [38]
**SIFT-MS**	CRLM (51); Controls (54)	0.28	0.89	Miller Atkins et al., 2020 [39]
**e-nose**	CRC (62) *	0.88	0.75	Steenhuis et al., 2020 [26]
**e-nose**	CRC (70); Controls (125)	0.95	0.64	Keulen et al., 2020 [27]
**SIFT-MS**	CRC (50); Controls (50) CRC (50); Controls (50) **	0.96 0.90	0.76 0.66	Markar et al., 2019 [40]
**e-nose**	CRC (15); Controls (15)	0.93	0.1	Altomare et al., 2016 [73]
**GC-MS + sensors**	CRC (65); Controls (122)	0.85	0.94	Amal et al., 2016 [8]
**GC-MS**	CRC (48); Controls (32) ***	1	0.98	Altomare et al., 2015 [41]
**GC-MS**	CRC (37); Controls (41)	0.86	0.83	Altomare et al., 2013 [43]

Note: CRLM: colorectal cancer liver metastases; GC-MS: gas chromatography—mass spectrometry; SIFT-MS: selected ion flow tube mass spectrometry; IMR-MS: ion molecule reaction–mass spectrometry. * Detection of extraluminal local recurrences or metastases in the follow-up of curatively treated CRC patients. ** Inflammatory bowel diseases (IBD), diverticular disease, and polyps. *** former Patients found disease free after oncologic follow up.

**Table 3:** Analysis pipelines of studies using breath-based VOCs towards CRC diagnosis.

Technique	Sample Size	Preprocessing Pipeline/Feature Extraction	Feature Selection	# of Features	Classifier	Validation	Validation Type	Reference
**Sensors**	CRC (105); Controls (186)	Data normalization; removal of erroneous sensor signals; extraction of statistical measures	Greedy stepwise selection; evolutionary search	75 (model with best results based on accuracy reported)	Random Forest, C4.5 (decision tree classifier); Artificial Neural Network; Naïve Bayes	70–30% split training and validation set	Internal	Poļaka et al., 2023 [72]
**GC-MS**	CRC (15); Controls (20)	Removal of artifacts; imputation using median; Shapiro–Wilk test;	Mann–Whitney U test; DFA; forward stepwise method; filtering based on certain metabolic reactions;	3	Artificial Neural Networks	10-fold Cross validation	Internal	Śmiełowska et al., 2023 [34]
**GC-MS**	CRC (30); Controls (84)	Noise removal; baseline correction; alignment; normalization; peak picking; scaling	Features detected in at least 20% of all classes considered	10	Isolation Forest	LOOCV	Internal	Cheng et al., 2022 [35]
**GC-MS**	CRC (162); Controls (1270)	Log-transformation; variance stabilization; normalization	ANOVA; Random forest	-	Random Forest, alphanet, SVM, LASSO; elastic net regression;	Repeated 5-fold Cross validation	Internal	Woodfield et al., 2022 [36]
**IMR-MS**	CRC (52); Controls (45)	Exclusion of specific chemicals (via *t*-test with reference sample); Standardization prior to modelling;	LASSO	15 (13 VOCs, Age, Sex)	Logistic Regression	50-fold Cross validation	Internal	Politi et al., 2021 [37]
**GC-MS + sensors**	CRC (82); Controls (87)	-	Mann–Whitney U test; univariate analysis and ranking of features; multivariate Stepwise Logistic Regression;	15 (14 VOCs, Age)	Logistic Regression	LOOCV	Internal	Altomare et al., 2020 [38]
**SIFT-MS**	CRLM (51); Controls (54)	Log-transformation; PCA noise removal; imputation of missing values (mean)	-	24 (22 VOCs, Sex, Age)	Random Forest	LOOCV on 95% of the dataset. 5% as a Test set	Internal	Miller Atkins et al., 2020 [39]
**e-nose**	CRC (62) *	TUCKER3	-	-	Artificial Neural Networks	10-fold cross validation	Internal	Steenhuis et al., 2020 [26]
**e-nose**	CRC (70); Controls (125)	Standardization; TUCKER3	-	11 (components derived from TUCKER3)	Artificial Neural Networks	10-fold cross validation	Internal	Keulen et al., 2020 [27]
**SIFT-MS**	CRC (50); Controls (50) CRC (50); Controls (50) **	-	Univariate statistics; Multivariate Logistic Regression	1	Logistic regression	100% of data as training set	Internal	Markar et al., 2019 [40]
**e-nose**	CRC (15);Controls (15)	Calculation of mean response of the signal	PCA based on variance explained	2 (1st and 2nd principal component)	Probabilistic Neural Networks	LOOCV	Internal	Altomare et al., 2016 [73]
**GC-MS + sensors**	CRC (65); Controls (122)	-	-	1 (Canonical variable from DFA applied to all sensing features)	DFA	70–30% split training and validation set	Internal	Amal et al., 2016 [8]
**GC-MS**	CRC (48); Controls (32) ***	-	Mann–Whitney U test	11	Probabilistic Neural Networks	LOOCV	Internal	Altomare et al., 2015 [41]
**GC-MS**	CRC (37); Controls (41)	-	Mann–Whitney U test	15	Probabilistic Neural Networks	LOOCV	Internal	Altomare et al., 2013 [43]

Note: GC-MS: gas chromatography—mass spectrometry; IMR-MS: ion molecule reaction–mass spectrometry; SIFT-MS: selected ion flow tube mass spectrometry; CRLM: colorectal cancer liver metastases; LOOCV: Leave one out cross validation; PCA: Principal Component Analysis; LASSO: Least absolute Shrinkage and selection operator; SVM: support vector machine. * Detection of extraluminal local recurrences or metastases in the Follow Up of curatively treated CRC patients. ** Inflammatory bowel diseases (IBD), diverticular disease, and polyps. *** former patients found disease free after oncologic follow up.

## Data Availability

This study did not generate or analyze any novel data; thus, data sharing does not apply.
